# The efficacy of fish oil in preventing coronary heart disease: A systematic review and meta-analysis

**DOI:** 10.1097/MD.0000000000027253

**Published:** 2021-09-17

**Authors:** Gaohong Wu, Qingyang Ji, Huiwen Huang, Xueping Zhu

**Affiliations:** a Department of Neonatology, Zhuhai Women and Children's Hospital, Zhuhai, China; b Department of Neonatology, Children's Hospital of Soochow University, Suzhou, China.

**Keywords:** coronary heart disease, fish oil, meta-analysis, prevention

## Abstract

**Background:**

Coronary heart disease (CHD) is one of the most common causes of death and disease burden in the world. Current fish oil aiming to prevent and treat CHD have shown a large variety of effects with low levels of evidence.

**Objective:**

To evaluate the efficacy of fish oil for protection against CHD, we conducted a systematic review and meta-analysis of randomized controlled trials (RCTs) evaluating the use of fish oil for protection against CHD.

**Methods:**

We retrieved relevant articles published from January 1966 to January 2020 by searching the PubMed, EMBASE, Cochrane CENTRAL, and Web of Science databases. RCTs of fish oil in preventing CHD were selected. The study quality was evaluated using the Cochrane Risk of Bias tool with RevMan 5.3 software. The first selection involved 360 citations. After screening and evaluation of suitability, 19 RCTs adjusted for clustering were included in the meta-analysis. All selected manuscripts considered that fish oil was effective in preventing CHD, secondary outcome measures included angina, sepsis and death.

**Results:**

Compared with the control group, fish oil may confer significant protection against CHD (odds ratio = 0.84; 95% confidence interval: 0.72–0.98). There was no significant difference in the incidence of secondary outcomes between the observation group and the control group (*P* > .05).

**Conclusion:**

The above results show that fish oil plays an important role in reducing CHD and cardiovascular events. However, because of the suboptimal quality of the studies included into the meta-analysis, these results do not justify adding fish oils systematically to the heavy pharmaceutical assortment already recommended in CHD patients.

**Registration details:**

CRD42020183719.

## 1 Introduction

Coronary heart disease (CHD) is one of the leading causes of death and disease burden in the world, the burden of disease is enormous both in the United States of America and around the world. In 2006, 18 million of the estimated 81 million adults in the America had CHD, and more than 400,000 Americans died of CHD. CHD is the cause of one in every 6 deaths in the America.^[1]^ While age-adjusted mortality rates appear to be falling globally, they remain high in low- and middle-income regions and countries, where there is a particularly high prevalence of risk factors.^[2]^ In developing countries such as China, the rising momentum of CHD burden has not been effectively curbed.^[3]^ Control of risk factors such as hypertension, diabetes, hyperlipidemia, obesity and smoking can reduce incidence and mortality rates of CHD.^[4]^ The use of antiplatelet drugs, statins, renin angiotensin aldosterone system blockers and β receptor blockers can also improve the prognosis of patients with CHD.^[5]^ Although efforts such as these have yielded some improvement in ameliorating CHD burden, a number of potential therapies that could be of further benefit remain to be fully explored. Recent years have seen growing interest in the notion that fish oil and ω-3 polyunsaturated fatty acids (PUFA) may be beneficial in the prevention and treatment of CHD, with a considerable number of studies have been carried out on the roles of fish oil and ω-3 PUFA in the clinical application of primary prevention and secondary prevention of CHD, including large-scale randomized controlled trials, multi center and single center cohort studies and observational studies.^[6]^ In this study, we conducted a comprehensive meta-analysis of the efficacy of fish oil for the prevention of CHD in order to establish its scientific basis, for the purposes of informing policies related to the use of fish oil.

## 2 Materials and methods

This work is a systematic review of published clinical studies. If necessary, meta-analysis will be possible. The data used in this systematic review will be all from published literature. Therefore, there is no need to provide ethical approval.

### 2.1 Application protocol and website recording data

A protocol including the investigation methods and the inclusion criteria for the current study was submitted in advance and documented on the center for review and dissemination York website PROSPERO, an international prospective register of systematic reviews. The parameters and the analytic structure of the present work can be viewed using the center for review and dissemination identification code: CRD42020183719.

This systematic investigation was conducted in accordance with the Preferred Reporting Items for Systematic Reviews and Meta-Analysis protocols.^[7]^

### 2.2 Search strategy

Articles published in English from January 1966 to January 2020 that explored the relationship between fish oil and protection against CHD were retrieved from PubMed, EMBASE, Cochrane CENTRAL, and Web of Science databases. The following search terms were used: “CHD,” “coronary heart disease,” “cardiovascular diseases,” “prevention,” and “fish oil”. Study design was randomized controlled trial (RCT), and the study was peer reviewed; the study population was human. Logical operators (OR, NOT, AND) were used to combine keywords and subject words. (Table 1).

**Table 1:** Search strings for the 4 databases.

Database	Search string
PubMed	(fish oil [MeSH Terms] OR ω-3 polyunsaturated fatty acids [Title/Abstract] OR Coronary heart disease [Title/Abstract] OR CHD [Title/Abstract] OR Cardiovascular diseases [Title/Abstract] OR prevention [Title/Abstract] OR control [Title/Abstract] OR measure [Title/Abstract] OR evaluate [Title/Abstract] OR effect [Title/Abstract] OR Health [Title/Abstract] OR Public health [Title/Abstract]
EMBASE	(‘Coronary heart disease’: ab, ti OR ‘CHD’:ab, ti) AND (‘Fish oil’:ab, ti OR ‘ω-3 polyunsaturated fatty acids’: ab, ti) AND (‘Health’:ab, ti OR ‘Public health’:ab, ti OR ‘Cardiovascular diseases’: ab, ti) AND (‘prevention’: ab, ti OR ‘control’: ab, ti OR ‘measure’: ab, ti OR ‘evaluate’: ab, ti OR ‘effect’:ab, ti OR ‘prevent’: ab, ti OR ‘control’: ab, ti OR ‘intervention’: ab, ti OR ‘outcome’:ab, ti)
Web of Science	TS = (Fish oil OR Fish OR ‘oil’ OR ‘ω-3 polyunsaturated fatty acids’ OR Coronary heart disease OR CHD OR ‘Cardiovascular diseases’ OR ‘prevention’ OR ‘control’ OR ‘prevention and control’ OR PPE OR ‘measur’ OR ‘evaluat’ OR ‘effect’ OR ‘Public’ OR ‘Public Healths’)
Cochrane CENTRAL	Coronary heart disease OR CHD in Title, Abstract, Keywords, AND ‘fish oil’ OR ‘fish’ OR ‘ω-3 polyunsaturated fatty acids’ in Title, Abstract, Keywords, AND practice OR control OR measure OR evaluate OR effect OR prevent OR prevention and control OR intervention OR outcome in Title, Abstract, Keywords, Publication Year from 1966 to 2020 in Trials

CHD = coronary heart disease.

### 2.3 Inclusion criteria

Articles that met the following criteria were selected: the exposure of interest was using fish oil; the outcome of interest was the proportion of fish oil use in the experimental and control groups; the main outcome measure was CHD. Secondary outcome measures included angina, sepsis and death. Studies took place in healthcare settings worldwide.

### 2.4 Exclusion criteria

The following exclusion criteria were applied: Trials in which patients were being treated with blood pressure disease, virus infected patients, osteoporosis, immunologic disorders, uncontrolled diabetes mellitus, or other surgical risk related systemic conditions; not enough information regarding the selected topic; trials that were not RCTs; no access to the title and abstract number in the English language.

### 2.5 Data extraction

Data extraction was conducted in 2 stages. First, literature was screened by 2 researchers according to inclusion criteria. The screened literature was then searched and evaluated by 2 other researchers according to inclusion and exclusion criteria. To avoid errors, a pre-designed form was used to select the study characteristics, baseline patient characteristics and outcomes and definitions included in the literature. Any inconsistencies in recommendations were resolved through consultation. The main data extracted were as follows: the number of people who were assigned to using fish oil and those who were not assigned to using fish oil.

### 2.6 Literature quality assessment

The quality of the methodology in the included studies was evaluated by using the Cochrane Risk of Bias tool.^[8]^ The quality of RCTs was evaluated using RevMan 5.3 software. The risk of bias was evaluated from 6 perspectives: choice bias, performance bias, measurement bias, attrition bias, reporting bias, other biases (Table 2). According to the criteria for low, unclear and high risk, the quality of the methodology of the included studies was divided into 3 levels: mild bias, where 4 or more of the above 6 items are low risk; moderate bias, where 2 or 3 of the above 6 items are low risk; and severe bias, where none or only one of the above 6 items is low risk.

**Table 2:** Cochrane risk of bias assessment form.

Evaluation items	Evaluation content	
Choice bias	Random sequence generation	The method of generating random assignment sequence is described in detail, which is convenient for evaluation of the comparability between groups.
	Assignment hidden	The method of hiding random distribution sequence is described in detail, which is convenient for judging whether the distribution of intervention measures can be predicted.
Performance bias	Blind method for researchers and subjects	The method of blinding used to prevent researchers and subjects from knowing the intervention measures is described in detail. This provides information that can be used to judge whether the blinding method is effective.
Measurement bias	Blind evaluation of research results	The method of blinding used to prevent the evaluators of the research results from knowing the intervention measures is described in detail. This provides information that can be used to judge whether the blinding method is effective.
Attrition bias	Integrity of result data	The data for each major outcome indicator, including those of subjects who were lost or withdrew from the study, are reported completely. Including subjects who were lost or withdrew, the total number of people in each group (compared with the total number of randomly enrolled people), and the reasons for the loss of interview/withdrawal are clearly reported, so as to facilitate assessment of the relevant treatment by the system evaluator.
Reporting bias	Selective reporting of research results	The information described can be used by system evaluators to judge the possibility of selective reporting of research results and relevant information.
Other biases	Other sources of bias	In addition to the above biases, the information provided can be used to assess the existence of other bias factors. If a question or factor is mentioned in the plan, corresponding answers are required.

### 2.7 Statistical methods

RevMan 5.3 software provided by the Cochrane Collaboration was used to conduct this meta-analysis of the proportions of fish oil use between the experimental and control groups. Q and *I*^2^ tests were used to evaluate the heterogeneity of the included studies (Q tests is the traditional method in the heterogeneity test of meta-analysis; *I*^2^ tests can measure the degree of difference among multiple research effects, and can describe the percentage of inter-research variation as a proportion of the total variation). When *I*^2^ ≤ 50% and *P* > .1, a fixed effect model was used to merge the data; when *I*^2^ > 50% or *P* < .1, a random effect model was used to merge the data. The odds ratio (OR) and 95% confidence interval (CI) were used to express the enumeration data. *P* < .05 was considered to indicate statistical significance.

### 2.8 Document retrieval flow chart (Fig. 1)

## 3 Results

### 3.1 Literature search results

**Figure 1:**
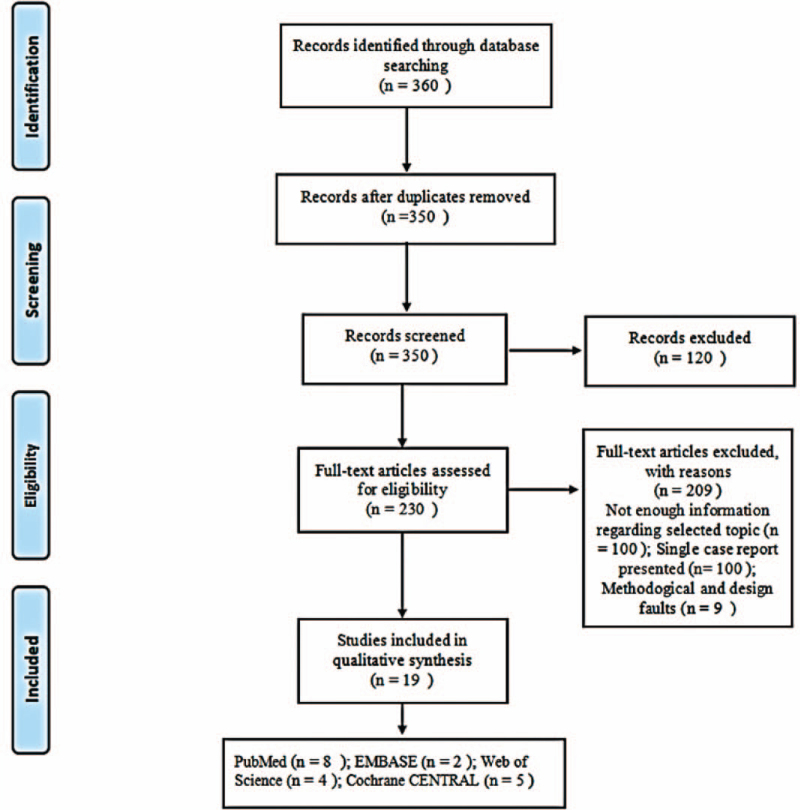
Summary of the literature search and inclusion process.

**Figure 2:**
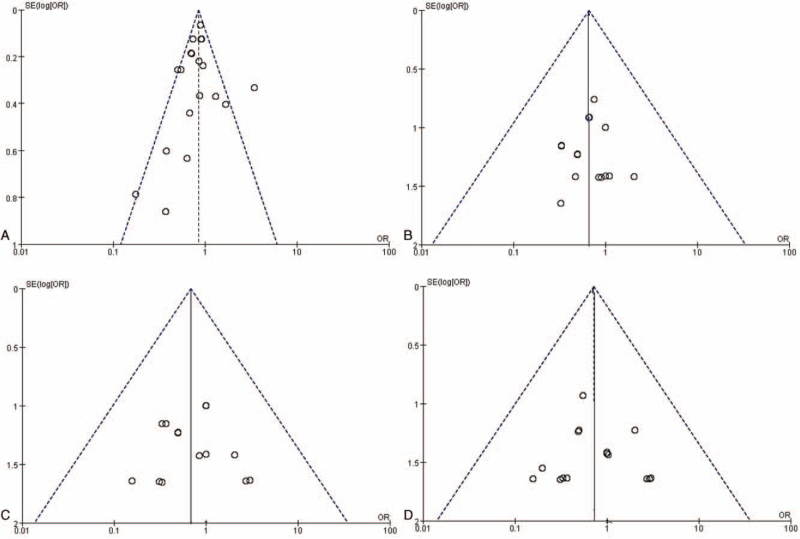
(A). RCTs effect of fish oil compared to no fish oil. Funnel plot assessing publication bias in RCTs investigating the effectiveness of fish oil for protection against CHD; Harbord's estimated bias coefficient: −0.44; *P* = .491. (B). RCTs effect of fish oil compared to no fish oil. Funnel plot assessing publication bias in RCTs investigating the effect of fish oil on death; Harbord's estimated bias coefficient: 0.40; *P* = .635. **C.** RCTs effect of fish oil compared to no fish oil. Funnel plot assessing publication bias in RCTs investigating the effect of fish oil on sepsis; Harbord's estimated bias coefficient: −0.58; *P* = .599. **D.** RCTs effect of fish oil compared to no fish oil. Funnel plot assessing publication bias in RCTs investigating the effect of fish oil on angina; Harbord's estimated bias coefficient: 0.41; *P* = .636. Funnel plots were generated to evaluate publication bias in RCT. The unadjusted effect estimates in some studies correspond to their standard errors. The real line and dotted line represent the aggregate effect estimates of different standard errors and their 95% CI, respectively. To determine publication bias, the Harbord test of small-study effects was used to assess funnel plot asymmetry.

After searching 360 papers from 4 databases, 19 articles were included in the final screening (Fig. 1). Of the 350 papers identified through database queries, we screened out 120, then searched the full texts of the remaining 230 articles, excluding 209 that did not meet our inclusion criteria, leaving 19 RCTs (Table 3). All of these RCTs analyzed the effectiveness of fish oil for protection against CHD. Moreover, they all analyzed the effect of fish oil on death, sepsis and angina. There was no real evidence to suggest publication bias (Fig. 2A, B, C, D)

**Table 3:** Summary of RCTs assessing the effectiveness of fish oil for protection against CHD (n = 19).

						Experimental group (n)				Control group (n)			
First author, year [reference number]	Journal	Study design	Continent	Blinding	Overall sample size	a	b	c	d	a	b	c	d
Borchgrevink, 1966 ^[9]^	Lancet	RCTs	Europe	Single-blind	200	10	1	1	0	14	2	0	0
Dayton, 1968 ^[10]^	Lancet	RCTs	North America	Double-blind	1702	53	1	0	0	71	3	0	0
MRC, 1968 ^[11]^	Lancet	RCTs	Europe	Single-blind	786	45	2	0	0	51	2	0	0
Leren, 1970 ^[12]^	Circulation	RCTs	North America	Single-blind	824	61	1	0	0	81	3	1	1
Turpeinen, 1979 ^[13]^	Int J Epidemiol	RCTs	Europe	Single-blind	922	25	1	1	1	47	1	1	1
Miettinen, 1983 ^[14]^	Int J Epidemiol	RCTs	Europe	Single-blind	714	27	2	1	1	46	3	3	2
Frantz, 1989 ^[15]^	Arteriosclerosis	RCTs	North America	Double-blind	18114	121	3	0	2	131	4	1	1
Burr, 1989 ^[16]^	Lancet	RCTs	Europe	Single-blind	4066	132	1	1	1	144	2	0	0
Reis, 1989 ^[17]^	Lancet	RCTs	North America	Single-blind	222	71	1	2	0	15	1	1	1
B-urr, 1989 ^[18]^	Lancet	RCTs	Europe	Single-blind	2133	127	1	0	0	180	1	0	1
Nye, 1990 ^[19]^	Aust N Z J Med	RCTs	Oceania	Single-blind	73	5	0	1	1	11	0	1	1
Watts, 1992 ^[20]^	Lancet	RCTs	Europe	Single-blind	110	2	0	0	1	5	0	0	2
Kaul, 1992 ^[21]^	Int J Cardiol	RCTs	Asia	Single-blind	107	26	1	1	2	16	1	1	3
Bellamy, 1992 ^[22]^	Eur Heart J	RCTs	Europe	Double-blind	120	31	0	0	1	33	1	0	1
Franzen, 1993 ^[23]^	Cathet Cardiovasc Diagn	RCTs	Europe	Double-blind	175	22	1	1	1	16	1	0	0
Sacks, 1995 ^[24]^	Am Coll Cardiol	RCTs	North America	Double-blind	80	5	0	0	2	7	0	1	1
Singh, 1997 ^[25]^	Cardiovasc Drugs Ther	RCTs	Asia	Double-blind	370	38	1	1	0	80	1	1	0
Von Schaky, 1999 ^[26]^	Ann Intern Med	RCTs	Europe	Double-blind	220	2	0	0	1	10	0	0	0
GISSI prevenzione trial, 1999 ^[27]^	The Cochrane Collaboration	RCTs	Europe	Single-blind	11524	459	2	2	0	509	3	2	2

CHD = coronary heart disease, RCTs = randomized controlled trials. a: CHD; b: Death; c: Sepsis; d: Angina.

### 3.2 Randomized controlled trials

**Figure 3:**
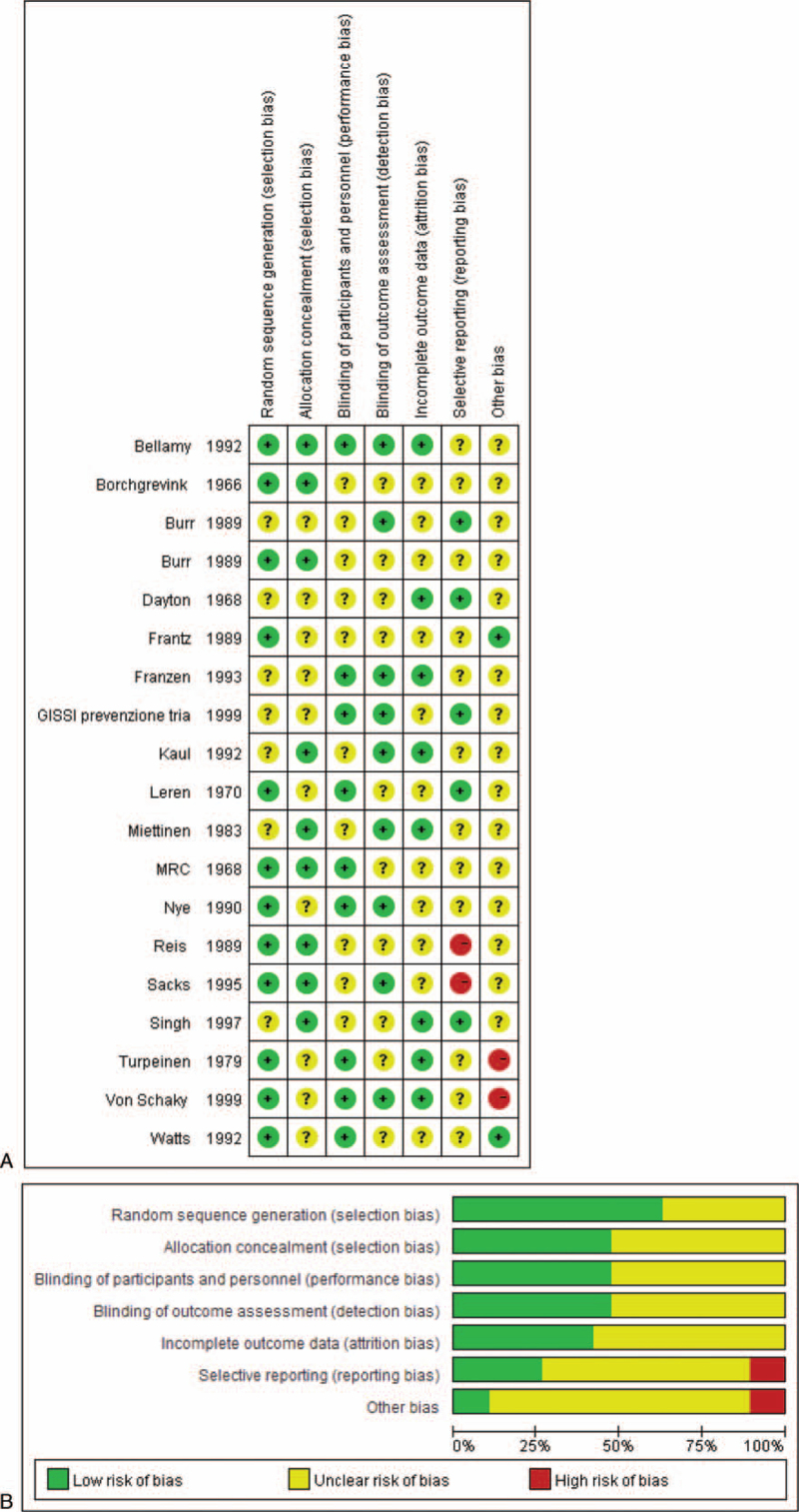
(A). RCTs received a high (red), low (green) or uncertain (yellow) risk of bias score for each of the domains. (B). Percentage of RCTs with high, low or uncertain risk of bias in each domain.

Assessment of the risk bias of 19 RCTs using RevMan 5.3 software showed moderate overall bias (Fig. 3A, B).

### 3.3 Fish oil use versus no fish oil use for protection against coronary heart disease

Nineteen RCTs compared CHD risk in people using fish oil to that of controls using no fish oil. Using fish oil conferred significantly greater protection against CHD (OR = 0.84; 95% CI: 0.72–0.98; *P* < .05) (Fig. 4 A). Because of heterogeneity, the data were divided for subgroup analysis according to the following: single-blind and double-blind; and Europe, North America and Asia. Subgroup analysis showed that heterogeneity of single-blind data was *I*^2^ = 67% (*P* = .0005) and the heterogeneity for double-blind was *I*^2^ = 6% (*P* = .38). This indicated that the heterogeneity of the double-blind data was much less than that of the single-blind data. Subgroup analysis by region showed that data heterogeneity for Europe was *I*^2^ = 32% (*P* = .14), for North America *I*^2^ = 79% (*P* = .0007), and for Asia *I*^2^ = 26% (*P* = .25), indicating that the North American data was far more heterogeneous than those of Europe and Asia. Therefore, the possibility that the heterogeneity of the data in the included studies was related to the type of blinding and continent could not be excluded (Fig. 4 B, C).

**Figure 4:**
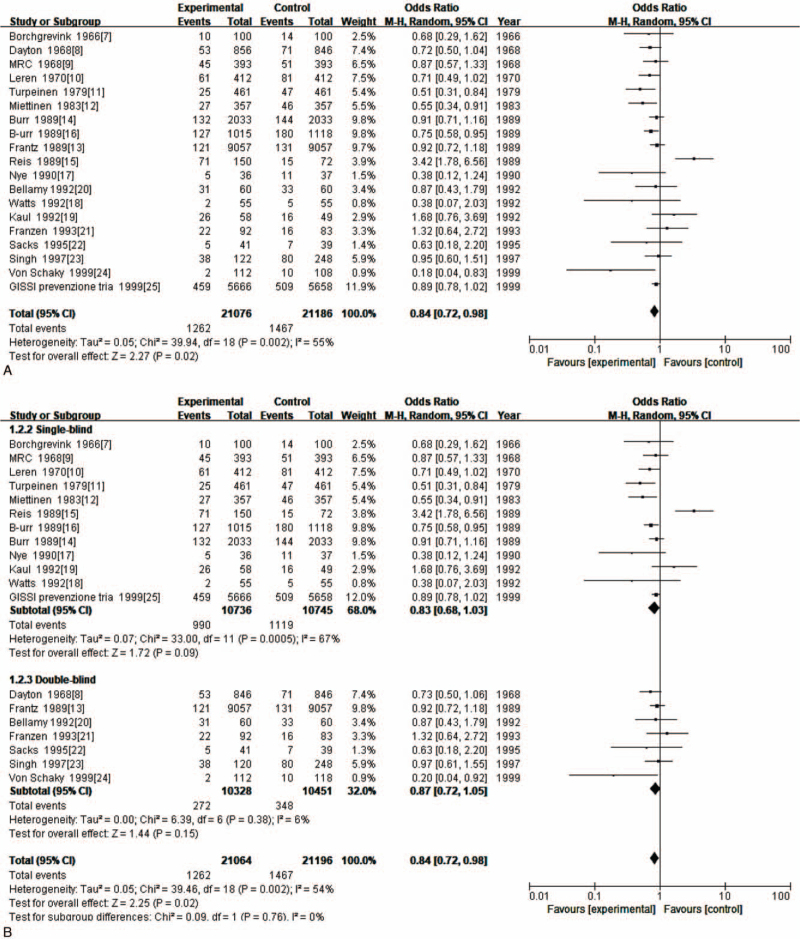
(A). Meta-analysis of the effect of using fish oil for protection against CHD. (B). Subgroup analysis of the effect of using fish oil for protection against CHD. (C). Subgroup analysis of the effect of using fish oil for protection against CHD.

**Figure 4 (Continued):**
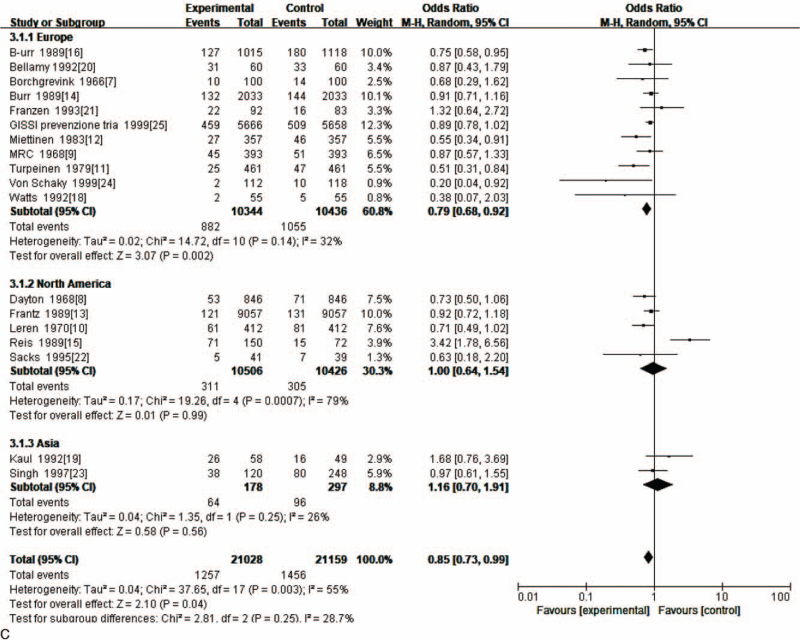
(A). Meta-analysis of the effect of using fish oil for protection against CHD. (B). Subgroup analysis of the effect of using fish oil for protection against CHD. (C). Subgroup analysis of the effect of using fish oil for protection against CHD.

### 3.4 The effect of fish oil on angina, sepsis, and death

Secondary outcome measures included death, sepsis and angina. Nineteen RCTs compared angina risk in people using fish oil to that of controls using no fish oil. There was no significant difference in the incidence of death between the observation group and the control group (OR = 0.65; 95% CI: 0.37–1.16; *P* > .05) (Fig. 5 A). There was no significant difference in the incidence of sepsis between the observation group and the control group (OR = 0.72; 95% CI: 0.38–1.37; *P* > .05) (Fig. 5 B). There was no significant difference in the incidence of sepsis between the observation group and the control group (OR = 0.71; 95% CI: 0.37–1.37; *P* > .05) (Fig. 5 C).

**Figure 5:**
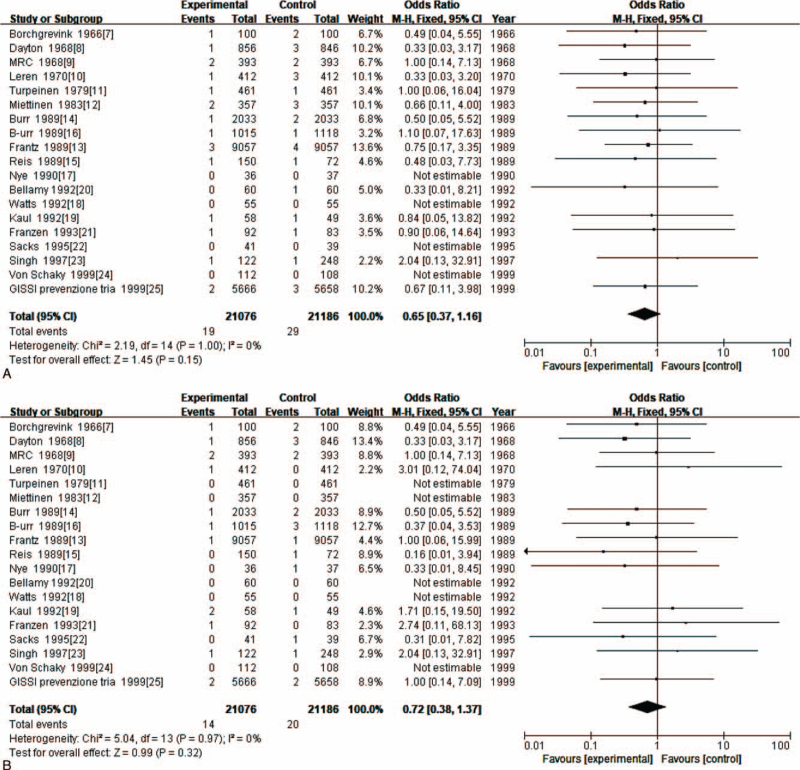
(A). Meta-analysis of the effect of fish oil on death. (B). Meta-analysis of the effect of fish oil on sepsis. (C). Meta-analysis of the effect of fish oil on angina.

**Figure 5 (Continued):**
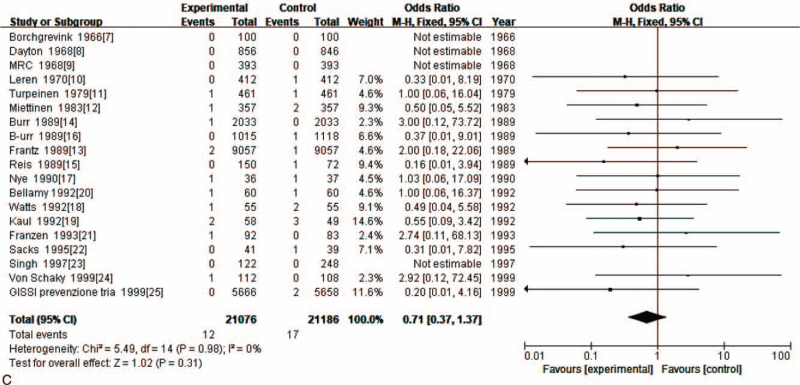
(A). Meta-analysis of the effect of fish oil on death. (B). Meta-analysis of the effect of fish oil on sepsis. (C). Meta-analysis of the effect of fish oil on angina.

## 4 Discussion

CHD is caused by coronary atherosclerotic plaque formation leading to vascular stenosis or obstruction caused by the supply area of myocardial ischemia, hypoxia or necrotic lesions. In recent years, diagnosis and treatment of CHD has been in the increasing trend especially for prevention.^[27]^ The clinical application of fish oil has attracted much research attention in recent years. In the present meta-analysis we sought to evaluate the evidence base on the efficacy of the use of fish oil in CHD. Fish oil is rich in ω-3 PUFA, the effects of which on cardiovascular metabolism are still under extensive study. ω-3 PUFA are essential – to stay healthy we must obtain some from food. The main types of ω-3 PUFA are alpha-linolenic acid, a fat found in plant foods, eicosapentaenoic acid and docosahexaenoic acid, both found in fish oil. There is a common belief that eating more fish oil reduces our risk of CHD, stroke and death.^[28]^ Preliminary studies have shown that ω-3 PUFA can reduce blood pressure, improve arterial elasticity, improve endothelial function, increase arrhythmia threshold, reduce platelet aggregation and improve autonomic nervous tension.^[29]^ However, Studies have also shown that Supplemental long-chain ω-3 PUFA are probably not useful for preventing or treating CHD.^[28]^ These inconsistent results may be related to the study design and researchers.

The results of the present meta-analysis of 19 RCTs show that the use of fish oil has a significant protective effect against CHD incidence when compared to no fish oil intervention.^[9–26]^ Some of the first suggestions of the putative relationship between dietary fish intake and CHD appear in the literature between 1985 and 1995.^[30–33]^ In 1996, Stone et al reviewed a number of prospective epidemiological studies, concluding that, compared with no fish oil consumption, eating fish oil can reduce mortality rate of patients with CHD.^[34]^ A subsequent retrospective analysis of physician-initiated health studies in 2002 found that basal plasma long chain ω-3 PUFA was significantly negatively correlated with sudden death.^[35]^ This study was divided into 4 groups according to plasma ω-3 PUFA levels. Compared with the lowest level group (group 1), the relative risk of sudden death in group 3 was 0.28, and that in group 4 was 0.19 (81% less). These findings are somewhat consistent with those of the present meta-analysis. However, we remain cautious in our interpretation due to the suboptimal quality of the studies included. The results we have obtained are too weak to justify adding fish oils systematically to the already heavy burden of pharmaceuticals prescribed to CHD patients. Moreover, no significant differences in death, sepsis and angina were found to be associated with fish oil use.

There were limitations to this meta-analysis. First, the number of included studies was small, which may have resulted in distribution bias. Analysis of a greater number of studies would be required to reduce the risk of distribution bias. Second, there may have been measurement bias, publication bias and selection bias in the included articles. Third, heterogeneity among the data in the included studies was identified, which may be related to the research population, region, and CHD subtypes. Although the subgroup analysis of the use of fish oil was conducted for some indicators in this study, it was not conducted for different populations or CHD subtypes. Therefore, more detailed subgroup analysis would be required to provide a more convincing basis for our conclusions. Finally, the source of CHD was not identified in all trials and some subjects may have been sicker than others before trial commencement.

## 5 Conclusions

Here, we conducted a literature review and meta-analysis of RCTs of the protective effects of fish oil against CHD. Our analysis provides some evidence to support the universal use of fish oil in the high risk CHD patient group. However, the evidence is not sufficiently strong to support the addition of fish oils to the already heavy pharmaceutical assortment given to CHD patients. In light of the evidence in this meta-analysis it would be appropriate to review official recommendations supporting supplemental fish oil intake.

## Acknowledgments

Thanks to the General Program of the National Natural Science Foundation of China (NSFC, No. 81771626; 81971423); The Jiangsu Provincial Maternal and Child Health Key Talents Project (No. FRC201731); Social Development project of Jiangsu Province (BE2020658); COVID-19 Infection emergency Technology project (SYS2020030).

## Author contributions

**Conceptualization:** Gaohong Wu, Huiwen Huang.

**Data curation:** Gaohong Wu, Qingyang Ji, Huiwen Huang.

**Formal analysis:** Gaohong Wu, Huiwen Huang.

**Funding acquisition:** Xueping Zhu.

**Investigation:** Gaohong Wu.

**Methodology:** Gaohong Wu.

**Project administration:** Gaohong Wu.

**Resources:** Gaohong Wu.

**Software:** Gaohong Wu.

**Supervision:** Gaohong Wu.

**Validation:** Gaohong Wu.

**Visualization:** Gaohong Wu, Huiwen Huang.

**Writing – original draft:** Gaohong Wu.

**Writing – review & editing:** Gaohong Wu, Huiwen Huang.

## References

[1] Lewis SJ. Lipid-lowering therapy: who can benefit? Vasc Health Risk Manag 2011;7:525–34. 21915170 10.2147/VHRM.S23113 PMC3166192

[2] Mondesir FL Levitan EB Malla G. Patient perspectives on factors influencing medication adherence among people with coronary heart disease (CHD) and CHD risk factors. Patient Prefer Adherence 2019;13:2017–27. 31819383 10.2147/PPA.S222176 PMC6890172

[3] Du X Patel A Anderson CS. Epidemiology of cardiovascular disease in china and opportunities for improvement: JACC international. J Am Coll Cardiol 2019;73:3135–47. 31221263 10.1016/j.jacc.2019.04.036

[4] Liu W Wang T Sun P. Expression of Hcy and blood lipid levels in serum of CHD patients and analysis of risk factors for CHD. Exp Ther Med 2019;17:1756–60. 30783445 10.3892/etm.2018.7111 PMC6364198

[5] Knopf H Busch MA Du Y. Secondary prevention of coronary heart disease in women and men in Germany from 1997–1999 and from 2008–2011-Trend analysis with two national health population surveys. Bundesgesundheitsblatt Gesundheitsforschung Gesundheitsschutz 2019;62:861–9. 31187183 10.1007/s00103-019-02975-1

[6] Zock PL Blom WA Nettleton JA. Progressing insights into the role of dietary fats in the prevention of cardiovascular disease. Curr Cardiol Rep 2016;18:111. 27650783 10.1007/s11886-016-0793-y PMC5030225

[7] Moher D Liberati A Tetzlaff J. Preferred reporting items for systematic reviews and meta-analyses: the PRISMA statement. J Clin Epidemiol 2009;62:1006–12. 19631508 10.1016/j.jclinepi.2009.06.005

[8] Michaelis R Tang V Wagner JL. Cochrane systematic review and meta-analysis of the impact of psychological treatments for people with epilepsy on health-related quality of life. Epilepsia 2018;59:315–32. 29313968 10.1111/epi.13989

[9] Borchgrevink CF Skaga E Berg KJ. Absence of prophylactic effect of linolenic acid in patients with coronary heart-disease. Lancet 1966;2:187–9. 4161161 10.1016/s0140-6736(66)92474-3

[10] Dayton S Pearce ML Goldman H. Controlled trial of a diet high in unsaturated fat for prevention of atherosclerotic complications. Lancet 1968;2:1060–2. 4176868 10.1016/s0140-6736(68)91531-6

[11] Medical Research Council. Controlled trial of soya-bean oil in myocardial infarction. Lancet 1968;2:693–9. 4175085

[12] Leren P. The Oslo diet-heart study. Eleven-year report. Circulation 1970;42:935–42. 5477261 10.1161/01.cir.42.5.935

[13] Turpeinen O Karvonen MJ Pekkarinen M. Dietary prevention of coronary heart disease: the Finnish Mental Hospital Study. Int J Epidemiol 1979;8:99–118. 393644 10.1093/ije/8.2.99

[14] Miettinen M Turpeinen O Karvonen MJ. Dietary prevention of coronary heart disease in women: the Finnish mental hospital study. Int J Epidemiol 1983;12:17–25. 6840954 10.1093/ije/12.1.17

[15] Frantz ID Dawson EA Ashman PL. Test of effect of lipid lowering by diet on cardiovascular risk. Minnesota Coronary Survey, Arteriosclerosis 1989;9:129–35. 2643423 10.1161/01.atv.9.1.129

[16] Burr ML Fehily AM Gilbert JF. Effects of changes in fat, fish, and fibre intakes on death and myocardial reinfarction: diet and reinfarction trial (DART). Lancet 1989;2:757–61. 2571009 10.1016/s0140-6736(89)90828-3

[17] Reis GJ Sipperly ME McCabe CH. Randomised trial of fish oil for prevention of restenosis after coronary angioplasty. Lancet 1989;2:177–81. 2568519 10.1016/s0140-6736(89)90370-x

[18] Nye ER Ablett MB Robertson MC. Effect of eicosapentaenoic acid on restenosis rate, clinical course and blood lipids in patients after percutaneous transluminal coronary angioplasty. Aust N Z J Med 1990;20:549–52. 2222347 10.1111/j.1445-5994.1990.tb01311.x

[19] Watts GF Lewis B Brunt JN. Effects on coronary artery disease of lipid-lowering diet, or diet plus cholestyramine, in the St Thomas’ Atherosclerosis Regression Study (STARS). Lancet 1992;339:563–9. 1347091 10.1016/0140-6736(92)90863-x

[20] Kaul U Sanghvi S Bahl VK. Fish oil supplements for prevention of restenosis after coronary angioplasty. Int J Cardiol 1992;35:87–93. 1563884 10.1016/0167-5273(92)90059-c

[21] Bellamy CM Schofield PM Faragher EB. Can supplementation of diet with omega-3 polyunsaturated fatty acids reduce coronary angioplasty restenosis rate? Eur Heart J 1992;13:1626–31. 1289091 10.1093/oxfordjournals.eurheartj.a060115

[22] Franzen D Schannwell M Oette K. A prospective, randomized, and double-blind trial on the effect of fish oil on the incidence of restenosis following PTCA. Cathet Cardiovasc Diagn 1993;28:301–10. 8462079 10.1002/ccd.1810280407

[23] Sacks FM Stone PH Gibson CM. Controlled trial of fish oil for regression of human coronary atherosclerosis. HARP Research Group. J Am Coll Cardiol 1995;25:1492–8. 7759696 10.1016/0735-1097(95)00095-l

[24] Singh RB Niaz MA Sharma JP. Randomized, double-blind, placebo-controlled trial of fish oil and mustard oil in patients with suspected acute myocardial infarction: the Indian experiment of infarct survival--4. Cardiovasc Drugs Ther 1997;11:485–91. 9310278 10.1023/a:1007757724505

[25] von Schacky C Angerer P Kothny W. The effect of dietary omega-3 fatty acids on coronary atherosclerosis. A randomized, double-blind, placebo-controlled trial. Ann Intern Med 1999;130:554–62. 10189324 10.7326/0003-4819-130-7-199904060-00003

[26] Mulrow C Oxman A. 10.7554/eLife.00857.037

[27] Zhang H Chang R. Effects of exercise after percutaneous coronary intervention on cardiac function and cardiovascular adverse events in patients with coronary heart disease: systematic review and meta-analysis. J Sports Sci Med 2019;18:213–22. 31191090 PMC6543998

[28] Abdelhamid AS Brown TJ Brainard JS. Omega-3 fatty acids for the primary and secondary prevention of cardiovascular disease. Cochrane Database Syst Rev 2018;7:CD003177. 30019766 10.1002/14651858.CD003177.pub3 PMC6513557

[29] de Bus I Witkamp R Zuilhof H. The role of n-3 PUFA-derived fatty acid derivatives and their oxygenated metabolites in the modulation of inflammation. Prostaglandins Other Lipid Mediat 2019;144:106351. 31260750 10.1016/j.prostaglandins.2019.106351

[30] Kromhout D Bosschieter EB de Lezenne Coulander C. The inverse relation between fish consumption and 20-year mortality from coronary heart disease. N Engl J Med 1985;312:1205–9. 3990713 10.1056/NEJM198505093121901

[31] Shekelle RB Missell L Paul O. Fish consumption and mortality from coronary heart disease. N Engl J Med 1985;313:820–4. 4033711 10.1056/NEJM198509263131311

[32] Gorder DD Dolecek TA Coleman GG. Dietary intake in the Multiple Risk Factor Intervention Trial (MRFIT): nutrient and food group changes over 6 years. J Am Diet Assoc 1986;86:744–51. 3519736

[33] Kromhout D Feskens EJ Bowles CH. The protective effect of a small amount of fish on coronary heart disease mortality in an elderly population. Int J E pidemiol 1995;24:340–5. 10.1093/ije/24.2.340 7635594

[34] Stone NJ. Fish consumption, fish oil, lipids, and coronary heart disease. Circulation 1996;94:2337–40. 8901708 10.1161/01.cir.94.9.2337

[35] Albert CM Campos H Stampfer MJ. Blood levels of long-chain n-3 fatty acids and the risk of sudden death. N Engl J Med 2002;346:1113–8. 11948270 10.1056/NEJMoa012918

